# *Blastomyces* Antigen Detection for Monitoring Progression of Blastomycosis in a Pregnant Adolescent

**DOI:** 10.1155/2007/89059

**Published:** 2007-05-23

**Authors:** Megan Tarr, John Marcinak, Kanokporn Mongkolrattanothai, Jennifer L. Burns, L. Joseph Wheat, Michelle Durkin, Mahmoud Ismail

**Affiliations:** ^1^Department of Obstetrics and Gynecology, University of Chicago, Chicago, IL 60637, USA; ^2^Section of Infectious Diseases, Department of Pediatrics, University of Chicago, Chicago, IL 60637, USA; ^3^Section of Infectious Diseases, Department of Pediatrics, University of Illinois, College of Medicine at Peoria, Peoria, IL 61637, USA; ^4^MiraVista Diagnostics and MiraBella Technologies, Indianapolis, IN 46241, USA; ^5^Section of Maternal Fetal Medicine, Department of Obstetrics and Gynecology, University of Chicago, Chicago, IL 60637, USA

## Abstract

Although disseminated blastomycosis is a rare complication in pregnancy, delay in diagnosis and treatment can be fatal. We investigate the use of the *Blastomyces* urine antigen in diagnosis following disease progression in the intrapartum, postpartum, and neonatal periods. We describe a case of disseminated blastomycosis in a pregnant adolescent and review the pertinent literature regarding treatment and monitoring blastomycosis in pregnancy and the neonatal periods. This is the first reported case in which the *Blastomyces* urine antigen is utilized as a method of following disease activity during pregnancy confirming absence of clinically evident disease in a neonate. Urine antigen detection for blastomycosis can be useful for following progression of disease in patients with disseminated blastomycosis in both the intrapartum and postpartum periods.

## 1. INTRODUCTION

*Blastomyces dermatitidis* is a dimorphic fungus endemic to the Ohio River and Mississippi River valleys and the Great Lakes Region. The fungus is found in soil, bird manure, and decaying vegetation. Inoculation is achieved through inhalation of the spores. Patients often report fatigue, a productive cough, weight loss, fever, and are hospitalized for presumptive bacterial pneumonia. The median number of days from exposure to onset of blastomycosis infection is 45 days [1]. Besides pneumonia, other clinical manifestations include cutaneous papular lesions, osteomyelitis, and frank fungemia; these latter manifestations are secondary to disseminated disease. Infection with *Blastomyces dermatitidis* can have a mortality rate ranging from 4.3% to 22% with higher mortality rates among those infected with HIV [1]. In pregnancy, cell-mediated immunity is impaired. This state of immunosuppression can facilitate the reactivation of the disease or the initial disease presentation [2].

Although spontaneous resolution of the disease can occur, the most common antifungal agent used in treatment of individuals is amphotericin B deoxycholate [1, 3]. Amphotericin B is also the drug of choice in pregnant women and has also been used to treat disseminated blastomycosis in pregnancy with no increased risks of adverse medication effects on the mother and no reports of teratogenicity in exposures in all three trimesters [3, 4].

## 2. CASE

A previously healthy 14-year-old African-American female was referred to the Orthopedic Surgery Clinic at University of Chicago Comer Children's Hospital (UCCH) by her primary physician for an evaluation of persistent low back pain. At the time of this visit in October 2003, she had a 4-week history of intermittent fever to 40°C, multiple skin lesions, fatigue, right 4th digit swelling, and a weight loss of 30 pounds. She was admitted to the intensive care unit for management of respiratory distress. On physical examination, she appeared to be short of breath and required oxygen due to low arterial oxygen saturation on room air (88%). She had a 2 cm ulcerative draining skin wound on the back of the left shoulder as well as 1 × 1 cm nodules over her arms, legs, and trunk. Her spleen was palpable 2 cm below the left costal margin. Her lungs were clear to auscultation.

Initial laboratory studies upon admission were significant for an elevation of aminotransferase enzymes (AST of 316 U/L and ALT of 332 U/L), erythrocyte sedimentation rate (129 mm/h), and c-reactive protein (140 mg/L). A chest radiograph showed a diffuse reticulonodular infiltrative pattern. Magnetic resonance imaging (MRI) of the thoracic and lumbar spine showed a paravertebral abscess at L5-S1 and osteomyelitis involving the T12, S1-S2 vertebral bodies and the right iliac bone.

A biopsy of a cutaneous nodule revealed broad-based budding yeasts on fungal smear that were morphologically consistent with *Blastomyces dermatitidis*. Sputum and skin biopsy cultures grew *Blastomyces dermatitidis. Blastomyces* antigen detection in urine was highly elevated at 37.69 EIA units (normal range <1 EIA units). Serologic assays (both complement fixation and immunodiffusion) for blastomycosis were negative. She was started on amphotericin B at 1 mg/kg/day for 2 weeks and switched to the oral itraconazole suspension 100 mg twice a day. The liver function tests improved gradually and were approaching normal before discharge. Prior to discharge, her back pain had resolved, and her respiratory status had normalized. An HIV antibody test was negative.

**Figure 1:**
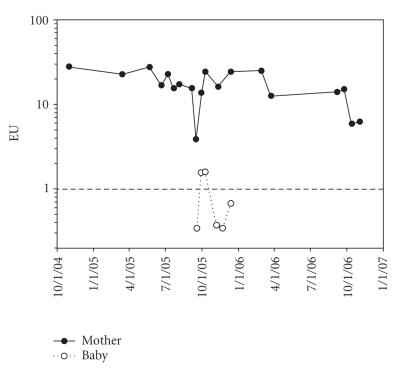
*Blastomyces* antigenuria during and after pregnancy for mother-baby pair.

In July 2004, she was readmitted to UCCH due to massive swelling of her right 4th digit. At that time, *Blastomyces* antigen detection in urine remained elevated at 28.77 EIA units. These findings raised the concern of nonadherence to itraconazole, which was supported by multiple failed clinic appointments. A plain radiograph of right hand showed findings consistent with osteomyelitis. After a consultation with the orthopedic surgery service, amputation of the right 4th finger was considered. Instead, intravenous amphotericin B at 1 mg/kg/day was initiated. A rise in serum creatinine (from 0.5 mg/dL to 2.5 mg/dL) occurred, and the antifungal regimen was changed to amphotericin B lipid complex. She demonstrated a remarkable decrease in swelling of the right 4th finger after beginning this regimen. Amphotericin B lipid complex was continued for 6 weeks followed by oral itraconazole capsules 100 mg twice per day. Surgery on the right fourth finger was unnecessary, and further MRIs of the spine showed resolution of the paravertebral abscess and decrease in signal intensity of the right sacral and iliac bones. Complement levels, lymphocyte subset levels, and IgA, IgM levels were normal. IgG levels were slightly elevated at 1722 mg/dL. She again was seen intermittently after the second hospitalization. When she returned to the pediatric infectious diseases clinic for followup in May 2005 (18 months after the initial diagnosis), she disclosed that she was 20-week pregnant. At this visit, the *Blastomyces* urine antigen remained elevated at 24.80 EIA units (Figure 1). Due to limited data concerning the use of itraconazole during pregnancy, which is classified as a category C medication, she was counseled regarding the possible risk to the developing fetus. Itraconazole was discontinued, and treatment with intravenous liposomal amphotericin B was recommended by the pediatric infectious disease service. Although further antifungal treatment was refused, she was closely followed in the pediatric infectious disease clinic with physical examinations and serial monitoring of the urine *Blastomyces* antigen test. The *Blastomyces* urine antigen remained stable but elevated throughout the third trimester of pregnancy (17.52–26.44 EIA units) up to 36-week gestation (Figure 1). The patient had one limited structural survey ultrasound performed at 17 weeks; results were unremarkable. Due to the patient's insurance status, the patient was not referred to the Maternal Fetal Medicine Department at the University of Chicago until 34-week gestation; she, however, delivered prior to her first visit. She had not received any antenatal testing.

The patient presented to the labor and delivery unit in active labor at term with intact amnionic membranes and immediately received one 400 mg (5 mg/kg) dose of the amphotericin B lipid complex. The patient also developed a fever 6 hours prior to delivery and was treated for chorioamnionitis in labor.

The patient delivered a healthy female infant weighing 2600g with APGARS of 9 at one and five minutes. The patient was restarted on oral itraconazole capsules 200 mg twice daily in the postpartum period. The mother had no further fever while hospitalized and was discharged on postpartum day number three. She received depomedroxyprogesterone acetate for contraception prior to discharge.

The placenta had no granulomas, and fungal culture from the placenta was negative. The infant's blood cultures at birth showed no growth, and urine and serum sent for *Blastomyces* antigen testing were also negative. A chest radiograph of the infant was also normal. At 2 and 3 weeks of life, the infant had a weakly positive urine *Blastomyces* antigen test, but subsequent urine samples sent up to age 12 weeks were negative (Figure 1).

Postpartum management included continuation of itraconazole capsules, 200 mg twice per day, biweekly monitoring of urine *Blastomyces* antigen levels, and monthly visits to the Pediatric Infectious Disease Clinic. *Blastomyces* urine antigen was persistently elevated for 5 months following delivery, and a measured serum itraconazole level was <0.3 *μ*g/mL (expected value- 0.3–7.0 *μ*g/mL). The itraconazole was changed from the capsule to the suspension form because of concern regarding absorption of the capsule formulation. Two weeks after the change in itraconazole formulation, a repeat itraconazole level drawn 1.5 hours after a direct observed dose was 2.4 *μ*g/mL. She was clinically well in follow-up while receiving antifungal therapy, and a *Blastomyces* urine antigen level measured 13 months after delivery has decreased to 6.22 EIA units (Figure 1).

## 3. COMMENT

Blastomycosis is an endemic mycosis in Southeastern, South central, and Midwestern states and is particularly prevalent in areas adjacent to the Mississippi and Ohio Rivers and the Great Lakes region. A recent Illinois Public Health Department epidemiological study of 500 cases of blastomycosis in Illinois reported no cases of blastomycosis in pregnant women over a ten-year period [1]. Although Mississippi is designated as the area with the highest prevalence of blastomycosis in the United States, studies from this region rarely report its incidence in pregnancy [2]. The clinical manifestations of blastomycosis during pregnancy are highly variable ranging from self-limited illness without treatment in some case reports [2] to disseminated infection [4], or acute respiratory distress syndrome [5].

Although fungal culture still remains the definitive diagnostic test, growth may be slow, which limits its value for early diagnosis. In contrast, antigen testing has the potential for rapid diagnosis of blastomycosis. The urine antigen test for blastomycosis has high sensitivity (92%) for the detection of the blastomycosis and excellent specificity (>98%) in patients without fungal infections, but is highly cross-reactive in those with histoplasmosis, paracoccidioidomycosis, and *Penicilliosis marneffei* [6]. Urine antigen detection of blastomycosis has recently been shown to be useful for followup during therapy of blastomycosis in pediatric patients, including the present adolescent patient prior to the pregnancy [7]. The effect of pregnancy on urine antigen for diagnosis of blastomycosis is not known, but our case illustrates that levels of antigen in the urine will persist if antifungal therapy is stopped or not taken consistently. Based on the experience with the urine antigen test for histoplasmosis [8], the urine antigen levels found in our adolescent are consistent with presumptive evidence of continued infection with blastomycosis.

It is of interest that our patient's level of urine *Blastomyces* antigen remained elevated in the 16–26 EIA unit range both during cessation of treatment during pregnancy and after reinitiation of treatment after delivery. The initial decrease in *Blastomyces* urine antigen levels occurred in the weeks following the short course of intravenous amphotericin B given during labor and while itraconazole was administered in the postpartum period. The decrease in urine antigen levels occurring a year after delivery indicates a successful response to antifungal therapy. The final outcome, however, remains to be determined. Our patient continues to receive antifungal therapy, and low-level antigenuria persists.

Blastomycosis can be transmitted from an infected pregnant woman to the newborn infant. The route of acquisition is not well established, and there are few reported cases. In a review of perinatal blastomycosis, transmission to the newborn infant was proposed to be through aspiration of colonized vaginal secretions as suggested by primarily pulmonary involvement in affected newborns and through hematogenous spread as evidenced by placental involvement [2, 9, 10]. There are no previous reports examining the use of *Blastomyces* urine antigen for diagnosis of newborn infants born to mothers with blastomycosis during pregnancy. In the past, diagnosis in the newborn has been through fungal culture, pulmonary cytology, or wet prep [2]. In our case, the infant did not develop any clinical manifestations of disease. This correlated with the transient, low *Blastomyces* urine antigen levels in the neonatal period that cleared with additional followup. Our case also illustrates that urine antigen detection for blastomycosis can be useful for following progression of disease in patient with disseminated blastomycosis in both the intrapartum and postpartum periods. Additional research is needed to determine the utility of the *Blastomyces* urine antigen test for diagnosis and monitoring disseminated blastomycosis.

## References

[1] Dworkin MS, Duckro AN, Proia L, Semel JD, Huhn G (2005). The epidemiology of blastomycosis in Illinois and factors associated with death. *Clinical Infectious Diseases*.

[2] Lemos LB, Soofi M, Amir E (2002). Blastomycosis and pregnancy. *Annals of Diagnostic Pathology*.

[3] Chapman SW, Bradsher WR, Campbell GD, Pappas PG, Kauffman CA (2000). Practice guidelines for the management of patients with blastomycosis. *Clinical Infectious Diseases*.

[4] Ismail M, Lerner SA (1982). Disseminated blastomycosis in a pregnant woman. Review of amphotericin B usage during pregnancy. *American Review of Respiratory Disease*.

[5] MacDonald D, Alguire PC (1990). Adult respiratory distress syndrome due to blastomycosis during pregnancy. *Chest*.

[6] Durkin M, Witt J, LeMonte A, Wheat B, Connolly P (2004). Antigen assay with the potential to aid in diagnosis of blastomycosis. *Journal of Clinical Microbiology*.

[7] Mongkolrattanothai K, Peev M, Wheat LJ, Marcinak J (2006). Urine antigen detection of blastomycosis in pediatric patients. *Pediatric Infectious Disease Journal*.

[8] Wheat LJ, Garringer T, Brizendine E, Connolly P (2002). Diagnosis of histoplasmosis by antigen detection based upon experience at the histoplasmosis reference laboratory. *Diagnostic Microbiology and Infectious Disease*.

[9] Maxson S, Miller SF, Tryka AF, Schutze GE (1992). Perinatal blastomycosis: a review. *Pediatric Infectious Disease Journal*.

[10] Watts EA, Gard PD, Tuthill SW (1983). First reported case of intrauterine transmission of blastomycosis. *Pediatric Infectious Disease*.

